# Chemical Structure of a Branched α-d-Glucan from the Eggs of Sea Urchin *Tripneustes gratilla*

**DOI:** 10.3390/ijms262110326

**Published:** 2025-10-23

**Authors:** Maria I. Bilan, Dmitry A. Argunov, Vladimir I. Torgov, Andrey S. Dmitrenok, Dinh Thanh Trung, Thinh Duc Pham, Hang Thi Thuy Cao, Anatolii I. Usov, Nikolay E. Nifantiev

**Affiliations:** 1 The Laboratory of Glycoconjugate Chemistry, N.D. Zelinsky Institute of Organic Chemistry, Russian Academy of Sciences, Leninsky Prospect 47, 119991 Moscow, Russia; bilan@ioc.ac.ru (M.I.B.); dm.argunov@gmail.com (D.A.A.); vladimir.torgov@mail.ru (V.I.T.); dmt@ioc.ac.ru (A.S.D.); usov@ioc.ac.ru (A.I.U.); 2 Institute of Oceanography, Vietnam Academy of Science and Technology, 01 Cau Da, Nha Trang 650000, Vietnam; dinhtrung@io.vast.vn (D.T.T.); ducthinh@io.vast.vn (T.D.P.); caohang.nitra@gmail.com (H.T.T.C.)

**Keywords:** sea urchin, *Tripneustes gratilla*, eggs, α-d-glucan, structure

## Abstract

A water-soluble high-molecular neutral polysaccharide (**NP**) was isolated from the eggs of the sea urchin *Tripneustes gratilla*. The formation of glucose only upon the treatment of **NP** by amyloglucosidase and the value of its optical rotation [α]_D_ +233.5 (c 0.2, water) confirmed its belonging to the family of α-d-glucans. According to the results of NMR spectroscopy and methylation analysis, the chains of **NP** are built up of non-reducing terminal, 4-linked and 4,6-disubstituted glucose residues at a ratio of 1:8:1. A branched structure with an average linear chain length of about five glucose residues was calculated from the spectrum of iodine complex. Contrary to the previously published structure of branched α-d-glucan from the sea urchin *Strongylocentrotus nudus* bearing single glucose units as branches, the polysaccharide **NP** contains oligosaccharide branches at position 6, which was confirmed by NMR data. Hence, **NP** has a glycogen-like structure with a rather high degree of branching, which markedly exceeds that of usual mammalian or fungal glycogens.

## 1. Introduction

Marine invertebrates belonging to the class Echinoidea of the phylum Echinodermata (known as sea urchins) contain several types of polysaccharides and glycoproteins. The most studied are sulfated fucans or galactans [1], components of so-called egg jelly, a thin layer composed of several proteins and bioglycans surrounding sea urchin eggs [2]. Structures of sulfated polysaccharides of egg jelly obtained from different species of sea urchins are usually species-specific. This feature explains the important biological role of sulfated polysaccharides capable of controlling the specificity of the very complex process of sea urchin fertilization [3,4]. Molecular structures of these polysaccharides can often be represented as regular polymers built up of identical repeating oligosaccharide fragments. Chemical structures of these repeating units can be completely characterized mostly using NMR spectroscopy [5]. The data obtained give important evidence on the correlations between structures and biological activities of sea urchin sulfated polysaccharides, known as anticoagulant and antithrombotic, as well as anti-inflammatory, antitumor, and antiviral agents [6,7,8,9]. At the same time, it is evident that the real practical application of components of sea urchin egg jelly in medicine is restricted by the limited availability of the natural sources.

Other tissues of sea urchins, such as intestine [10] or even shells [11,12], may contain different polysaccharides, but the richest sources are gonads or eggs, where appreciable amounts of proteins and polysaccharides are stored and used as reserve materials in the process of gametogenesis [13]. Thus, a water-soluble polysaccharide (SEP) was isolated from the eggs of *Strongylocentrotus nudus* and investigated by chemical and spectroscopic analytical methods [14]. It was found to be a branched α-d-glucan with a (1→4)-linked backbone with branches at C-6 (one branch at every nine residues). The authors suggested that these branches are single α-d-glucose residues, but the structure of the branches was not really investigated. This glucan has a rather high weight-average molecular weight of about 1.95 × 10^6^ Da. It could inhibit the growth of Sarcoma 180, acting probably by an immunomodulation mechanism. Later, the biological activity of SEP was investigated more thoroughly and described in several subsequent papers [15,16,17,18,19]. In addition to SEP, another α-glucan (SEP-2), also with immunostimulatory activity, was isolated from aqueous extract of *S. nudus* eggs by resolution of SEP and SEP-2 using anion-exchange chromatography [20]. According to the data from methylation analysis, SEP-2 contained 3- and 4-linked glucose residues in linear chains and 3,4-disubstituted residues as branching points. Distribution of differently substituted glucose residues between backbone and branches was not investigated, and the cause of retention of SEP-2 on the anion-exchanger was not explained.

A very similar to SEP highly branched α-glucan (MSGA) was isolated from the eggs of *S. intermedius*, the species belonging to the same genus as *S. nudus* [21]. The polysaccharide with a MW of 2.65 × 10^7^ Da was shown to be built up of 4-linked Glc in linear chains and 4,6-linked Glc at branching points, having one branch per five residues (data of methylation analysis). The authors termed MSGA as glycogen in the title of the paper and suggested the conventional glycogen-like branched structure for its chains, although the real distribution of glucose residues between backbone and side chains was not determined. MSGA showed good immunostimulatory activity, and a high degree of branching was suggested to be important for this feature. It is interesting to add that the presence of another polysaccharide component (SUP) in the gonads of *S. intermedius* was described previously [22]. SUP was isolated in a rather small yield after destruction of the main glycogen-like component by amylolysis. MW of SUP was only 4.4 kDa, the polysaccharide was composed mainly of 3-, 4-, and 6-linked mannose residues with unexpectedly high non-reducing terminal Man content, and also contained some glucose, glucosamine, and sulfate. The molecular formula suggested by the authors [22] is rather ambiguous. SUP was biologically active, since it can promote bone regeneration due to synergism with bone morphogenetic protein-2 (BMP-2).

A new branched α-glucan (HPP-1S) with a molecular weight of 2.996 × 10^7^ Da was isolated from the gonads of *Hemicentrotus pulcherrimus* [23]. It contained, in addition to glucose, also 6.2% of glucuronic acid. According to analytical data, the authors suggested the presence in HPP-1S a linear backbone of 4-linked α-d-Glc, some of which carried α-d-Glc or α-d-GlcA at position 6 as side substituents. HPP-1S exhibited obvious antitumor activity with low toxicity in both in vitro and in vivo experiments. Further investigation of the same source [24] resulted in the isolation of a series of α-glucan homologues (HPP-2S–HPP-8S), having molecular weights similar to HPP-1S and differing mostly in degree of branching. The polysaccharides with a higher degree of branching exhibited markedly enhanced immunomodulatory capacity.

Storage polysaccharides can often be obtained in satisfactory yields without difficulties due to their high content in numerous sources. According to the chemical structure, the majority of animal storage polysaccharides belong to the glycogen family. They are built up of three types of α-d-glucopyranose residues, namely, non-reducing terminals, 4-linked components of linear chains, and 4,6-disubstituted residues serving as branching points. This type of structure was ascribed to the great number of polymeric molecules differing in origin, molecular mass, degree of branching, and distribution of branching points along the linear chains. Additional structural details, such as alternative glycosidic bonds or additional monosaccharides, may be found in storage polysaccharides of some exotic animals, like, for example, sea urchins. All the listed structural features may have a great influence on the biological properties of polysaccharides. Thus, as mentioned above, pharmacological studies revealed that SEP from *S. nudus* eggs could inhibit the growth of Sarcoma 180 in vivo and stimulate the spleen lymphocyte proliferation in S180-bearing mice [14]. Further articles gave additional evidence on the immunomodulatory and anticancer activity of SEP [15,16,17,18,19], as well as on its potential to ameliorate inflammatory bowel disease [19]. Another polysaccharide from the same species (SEP-2) was also shown to have immunostimulatory properties, since it significantly enhanced ROS level, NO production, and inflammatory cytokine secretion (IL-1β, IL-6, and TNF-α) in the SEP-2-treated murine macrophage cell line RAW264.7 [20]. Promising anticancer and immunomodulatory activities were also detected in structurally related glucans isolated from *S. intermedius* [21,22] and *H. pulcherrimus* [23,24].

In order to enlarge the knowledge on the sea urchin polysaccharides, we have analyzed the polysaccharide composition of the eggs of *Tripneustes gratilla* (Linnaeus, 1758). This species is widely distributed in the Indo-Pacific region and was collected from Vietnamese coastal waters. A neutral α-glucan (**NP**) was isolated from the eggs with a rather high yield, and the glycogen-like structure of **NP** was characterized by chemical methods and NMR spectroscopy.

## 2. Results

### 2.1. Polysaccharide Isolation

Eggs of *T. gratilla* were obtained in Nha Trang according to conventional procedures, dehydrated, and the dry preparation was delivered to Moscow for composition analysis. The biomass was defatted, suspended in water, and treated with papain at a slightly elevated temperature in order to destroy proteins and to solubilize polysaccharides. Addition of Cetavlon (hexadecyltrimethylammonium bromide) to the aqueous extract resulted in the formation of negligible precipitate, which was separated by centrifugation and not investigated further. Supernatant was dialyzed, concentrated, and mixed with 4 volumes of ethanol. The precipitate formed was dissolved in water and lyophilized, giving rise to fraction **NP** of neutral polysaccharides, composed mainly of glucose (95.7%) with traces of mannose (1.0%). Action of amyloglucosidase on **NP** resulted in the formation of glucose with a yield of 74.2% (for comparison, 79.9% of glucose was obtained under the same conditions from commercial amylopectin). Compositional analysis (Table 1) confirmed high purity of NP. Our GPC system could not be used for the determination of very high MW of **NP**, but a product of its partial degradation by mild acid treatment **NP-H** showed a symmetrical peak corresponding to about 800 kDa, which demonstrates high homogeneity and absence of impurities in the preparation (Figure S1).

### 2.2. Methylation Analysis

Methylation analysis of **NP** showed the presence of terminal, 4-linked, and 4,6-disubstituted glucose residues at a ratio of 1:8:1 (cf. 4:91:5 obtained for commercial amylopectin) (Table 2), but, of course, gave no evidence of the distribution of these three types of residues between the main backbone and side chains.

### 2.3. NMR Analysis

**Figure 1:**
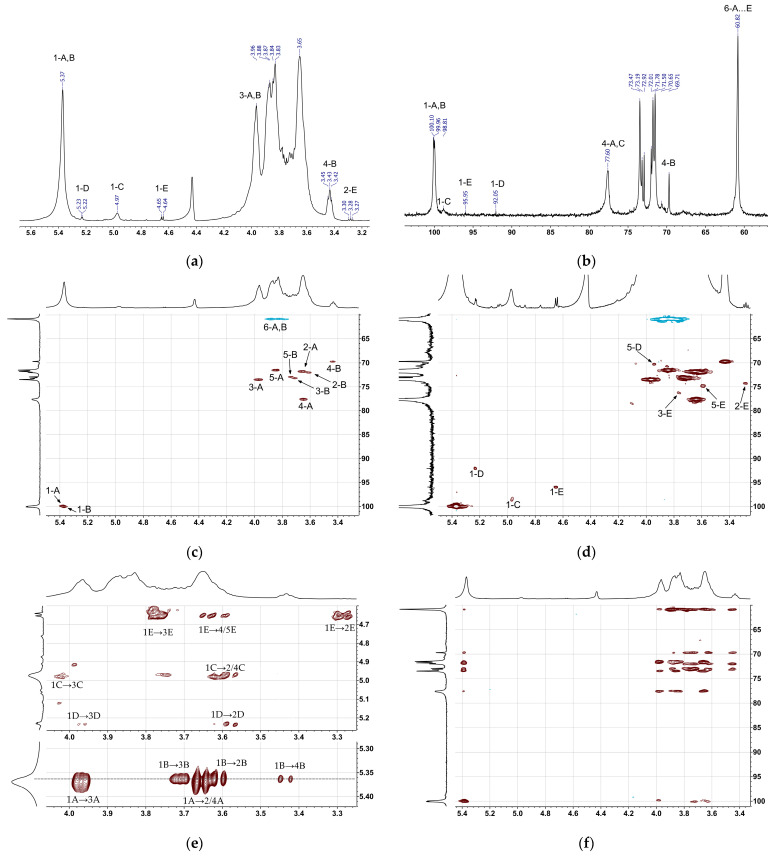
NMR spectra of native polysaccharide **NP** in D_2_O: (**a**) ^1^H (with solvent presaturation); (**b**) ^13^C; (**c**) [^1^H-^13^C]-HSQC; (**d**) [^1^H-^13^C]-HSQC with lower first contour level limit; (**e**) [^1^H-^1^H]-TOCSY; and (**f**) [^1^H-^13^C]-HSQC-TOCSY.

In addition to the methylation data, NMR spectra of **NP** were recorded and analyzed (Table 3, Figure 1). Theoretically, the equally substituted but differently arranged glucose residues should have slightly different NMR spectra, which might be used to characterize the size of side chains in **NP**. Such spectral differences were really observed by detailed spectral analysis of many branched low-molecular amylodextrins [25,26]. Unfortunately, the very high molecular weight of **NP** (approximately 2 × 10^7^ Da) hampers obtaining the satisfactory resolution of signals, and all attempts to improve quality of the spectra by partial degradation of polysaccharide by mild acid treatment, oxidation by hydrogen peroxide, or ultrasonication were rather ineffective. Nevertheless, it was found that ^1^H and ^13^C NMR spectra of **NP** revealed a typical pattern for branched α-(1→4)-glycans containing 4,6-branches (Figure 1a,b). The most intense correlations in the HSQC spectrum were attributed to the linear α-(1→4) chains according to a significant downfield shift in C-4 and H-4 signals (Table 3, unit A). High-field triplet in ^1^H spectrum at 3.43 ppm was attributed to H-4 of non-reducing terminal glucose residues. TOCSY and HSQC-spectra assignments starting from this signal unambiguously lead to the conclusion that these terminal residues are connected exclusively to O-4 (and not to O-6) of neighboring glucose units (Table 3, unit B). Minor anomeric signal at 4.96/98.81 ppm was attributed to units, connected to O-6 positions on the basis of its significantly high-field shift (unit C) [26]. Correlations in TOCSY from H-1 showed only O-4 substituted positions in the units, connected to O-6. In both reducing end residues D and E, only patterns for O-4 glycosylated units were observed.

Signals of 4,6-branched units were not sufficiently resolved in ^13^C-spectra due to the lack of main chain mobility in solution, high molecular weight, and the branched nature of the polysaccharide. However, on the basis of typical chemical shifts for model compounds, we might suggest that the broad signal at 70.65 can be attributed to C-5 of units carrying branches at C-6, whereas the broad baseline hump at 67.9 ppm corresponds to substituted C-6 atoms [26]. Rather significant high-field shift in C-5 from the disubstituted unit, as well as all the other ^13^C chemical shift assignments, were also in agreement with ones generated by the empirical NMR simulation tool of the Carbohydrate Structure Database (CSDB) [27,28,29,30].

Surprisingly, in many previous works, describing related polysaccharides isolated from bacteria, fungi, or higher plants and featuring similar structures and almost identical spectra, the authors claimed the presence of only single monosaccharide residues at branching points without providing enough evidence supporting this statement [14,31,32,33,34,35,36,37]. Several authors assumed the presence of single Glc side by side with the usual (1→4)-linked side chains [38], but this finding was also not unambiguously proved. Data [39] on short (1→6)-linked oligosaccharide branches, structurally related to dextrans, is evidently explained by the admixture of Sephadex, which was used as sorbent in the isolation procedure [38]. Analyzing the NMR spectra of **NP**, we have not observed TOCSY correlations from H-4 of unsubstituted position 4 to H-1 of residue C, which contradicts the conclusion about the presence of monosaccharide branches in **NP**.

Alongside disputable claims about monosaccharide-long side chains, we were also unable to observe signals of 4,6-branched units in the NMR spectra provided in these papers. Weak C-6 signal of 4,6-branched residue was only observed in the ^13^C-DEPT spectrum of alkali extract from *Pseudallescheria boydii* [40]. Notably, authors explicitly claimed that no other signals from this type of unit can be traced in NMR spectra. Significant inconsistencies in disubstituted residue chemical shifts between this work and data from oligosaccharides only reinforced our doubts. Low prediction accuracy of CSDB GODESS NMR simulations based on publications [14,31,32,33,34,35,36,37] additionally supports the observed low quality of assignments.

### 2.4. Analysis of Iodine Complexes

Iodine complexes of glycogens and amylopectins have different staining properties depending on the average chain length (ACL) distribution [41], and determination of the wavelength of maximum absorption (λ_max_) of these complexes was recommended to characterize the ACL of branched (1→4, 1→6)-α-D-glucans [42]. In our experiments, λ_max_ of iodine complexes of authentic glycogen and amylopectin were 472 and 559 nm, corresponding to the usual ACL of 13 and 23 glucose residues, respectively. In contrast, **NP** in standard conditions gave an iodine complex that had a very low intensity with a non-specific λ_max_ value of 403 nm corresponding to an ACL of 5. This behavior may be explained by a very high degree of branching of **NP**, resembling, for example, a highly branched storage glucan of the thermoacidophilic red microalga *Galdieria maxima* [43,44].

## 3. Discussion

Glycogens are storage glucose polymers found both in eucaryotes and in bacteria [45]. Native glycogens are branched biopolymers built up of 4-linked α-d-Glc forming linear chains, where several 4,6-disubstituted Glc residues carry oligosaccharide branches. Glycogens obtained from different organisms or tissues may differ considerably in molar mass distribution, average linear chain length, and degree of branching. Typically, the average linear chains of mammalian glycogens contain approximately 10–14 glucose units. Disruptions in the biosynthetic processes result in various glycogen-storage diseases, characterized by an unusual structure of polysaccharide or its abnormal accumulation in tissues [46]. Glycogens with short average chain length were found in bacteria, which is probably important for enhancing bacterial durability [47].

Marine invertebrates may be regarded as a rich source of glycogens. Supposedly, polysaccharides obtained from such exotic sources may have unusual structural features, resulting in some new types of biological activity. As mentioned in the Introduction, such a glycogen-like polysaccharide was isolated from the eggs of the sea urchin *Strongylocentrotus nudus* [14] and showed several interesting actions as an immunomodulator [15,16,17,18,19,20]. The molecular formula suggested for this polysaccharide was based on the data of methylation analysis, but the supposed presence of only a single Glc as branches was not confirmed. In our work, we obtained a very similar polysaccharide **NP** from another sea urchin species, *Tripneustes gratilla*, and characterized its branched structure by methylation, NMR spectroscopy, and a spectrum of iodine complex. According to these data, **NP** contains no branches in the form of single glucose residues. The absence of monosaccharide branches coincides with the current evidence on the biosynthesis of glycogens, where oligosaccharide side chains are generated by the action of branching enzymes [45].

**Scheme 1:**
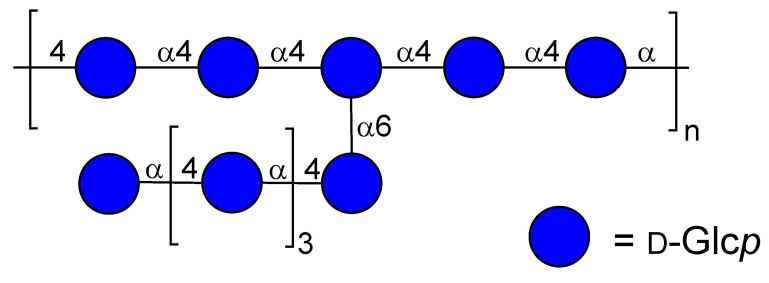
Proposed structure of **NP**.

Combining data of methylation analysis, NMR spectra, and characteristics of iodine complex, a glycogen-like structure was suggested for **NP** (Scheme 1), where oligosaccharide side chains of about five glucose residues in length are linked to every fifth residue of the backbone. Thus, this product can be used as a model for further investigation of immunomodulatory and other biological activities of natural α-glucans and related oligoglucosides [48]. The biological activity of NP is being studied now in several experiments, and the results will be published elsewhere.

## 4. Materials and Methods

### 4.1. Materials

**Figure 2:**
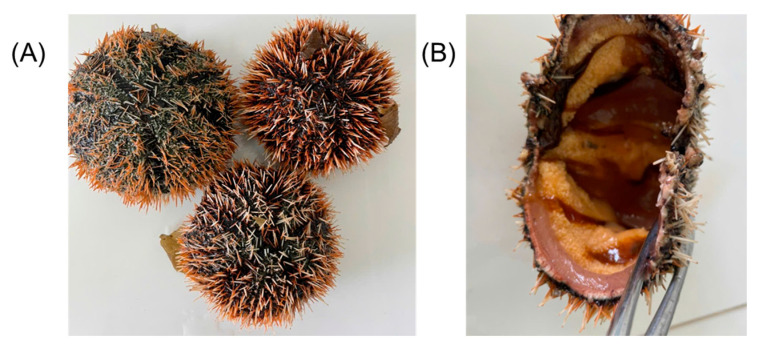
Morphological characteristics of the sea urchin: (**A**) Whole specimens showing short, dense spines with distinctive orange and dark bands, and (**B**) dissected individual displaying the characteristic orange gonads.

Sea urchins *Tripneustes gratilla* were collected from Nha Trang Bay, Vietnam, in November 2022 (12°16′17.94″ N; 109°12′26.13″ E). The identification of the sea urchin species was performed morphologically by Dr. Nguyen An Khang, Institute of Oceanography (Vietnam Academy of Science and Technology), following standard taxonomic keys [49]. Diagnostic characters included the hemispherical test with a diameter of 8–10 cm, short and dense spines with distinctive orange and dark bands, and the characteristic orange gonads. Representative photographs of whole specimens and dissected individuals are provided to illustrate these features (Figure 2).

The gonads obtained by dissecting adult sea urchins were opened, washed carefully with filtered seawater, and the suspension of eggs was centrifuged at 3000 rpm, 15 °C for 15 min; the precipitated eggs were lyophilized using a freeze dryer (Harvest HR-02, Salt Lake City, UT, USA). Samples were first frozen at −50 °C and then subjected to a drying cycle for 48 h under reduced pressure. During the drying process, the shelf temperature was maintained at approximately −10 °C to facilitate sublimation while preventing thermal degradation of the samples. Amyloglucosidase was from Total Starch Kit of Megazyme Int. (Wicklow, Ireland) Potato amylopectin and rabbit muscle glycogen were from Gee Lawson Chemicals Ltd. (London, UK). Standard monosaccharides and standard MW pullulans were purchased from Sigma (St. Louis, MO, USA). All chemicals used were of analytical grade or above and were used without further purification.

### 4.2. General Methods

Quantitative determination of monosaccharides by gas–liquid chromatography of alditol acetates was carried out as described previously [50,51]. Polysaccharide samples were hydrolyzed with 2 M trifluoroacetic acid (containing myo-inositol, 1 mg/mL, as internal standard) at 100 °C for 8 h. A turbidimetric procedure was used for determination of sulfate [52] after hydrolysis of polysaccharides, as mentioned above. Uronic acids were estimated colorimetrically with 3,5-dimethylphenol and sulfuric acid [53], using glucuronic acid for calibration. Protein was estimated by Lowry procedure [54], and calibration was carried out with solutions of BSA.

Gas chromatography was performed on an Agilent 8860 GC system (Santa Clara, CA, USA) equipped with a flame-ionization detector and an HP-5 capillary column (0.25 μm × 0.32 mm × 30 m). Spectral measurements were carried out using the spectrophotometer Spectronic Genesis 5 (Rochester, NY, USA). Optical rotations were measured using a JASCO P-2000 polarimeter (Easton, MD, USA) at ambient temperature (22–25 °C).

### 4.3. Isolation of NP

Dry powder of sea urchin eggs (16 g) was treated three times with a mixture of methanol, chloroform, and water (4:2:1) [55]. Defatted material was suspended in 300 mL of 0.1 M sodium acetate, pH 6, containing 5 mM EDTA and 5 mM l-cysteine hydrochloride, and 3 g of papain were added, and the mixture was incubated for 24 h at 60 °C [56]. Then, the mixture was heated for 20 min in a boiling water bath to inactivate the enzyme, cooled, centrifuged, and a 10% aqueous cetyltrimethylammonium bromide solution was added to complete precipitation of acid polysaccharides. A small precipitate (67 mg) separated by centrifugation was discarded. Supernatant was dialyzed against distilled water for 4 days at room temperature, using a cellulosic membrane (3500 Da cut off), concentrated and treated with 4 volumes of ethanol; the precipitate was separated, dissolved in water, and lyophilized to obtain **NP**, with a yield of 2 g, composition: Glc 96.7%, Man 1.0%. Tests for uronic acids, protein, and sulfate were negative.

### 4.4. Partial Degradation of NP

A solution of **NP** (197 mg) in 0.1 M HCl was incubated at 50 °C for 2 h, cooled, neutralized with NaOH, and desalted on a column with Sephadex G-15 to obtain an acid-degraded preparation **NP-H**, yielding 177 mg, [α]_D_^23^ +233.5 (c 0.2; water). According to GPC, **NP-H** practically coincided in mobility with standard pullulan with a MW of 805 kDa.

### 4.5. Molecular Weight Determination

Determination of the molecular weights was carried out using gel chromatography on a tandem column that contained TSK gel G 3000 SW, 7.8 × 300 mm, and TSK gel G 5000 PWXL, 7.8 × 300 mm. A pump Gilson 305 (Villiers Le Bel, France) was used for elution with a solution of 10 mM Na_2_HPO_4_, 150 mM NaCl in water, pH 7.5, at 0.5 mL/min. Refractometer Knauer (Berlin, Germany), was used as a detector. Standard pullulans with a MW 805, 348, 200, 48.8, 23, 10, and 6.2 kDa were used for calibration.

### 4.6. Methylation Analysis

The experimental procedure was conducted as previously described [57,58]. Briefly, 5 mg of **NP** was suspended in 0.5 mL of DMSO, and a finely ground NaOH (30 mg) and CH_3_I (0.2 mL) were added, and the mixture was stirred for 1 h at room temperature (an additional 0.2 mL of CH_3_I was added after 30 min). Then, water (4 mL) and chloroform (4 mL) were added, the mixture was dialyzed, evaporated to remove chloroform, and lyophilized. The methylated product was then hydrolyzed with 2 M trifluoroacetic acid (1 mL) at 100 °C for 8 h. Subsequently, the hydrolysate was reduced with NaBH_4_ and acetylated with acetic anhydride in the presence of pyridine, yielding partially methylated alditol acetates, which were analyzed by GLC. Authentic amylopectin was methylated and analyzed for comparison.

### 4.7. NMR Spectroscopy

NMR spectra were recorded in the Shared Research Center (Department of Structural Studies) of N.D. Zelinsky Institute of Organic Chemistry RAS, Moscow, on Bruker Avance II 600 (600 MHz) (Karlsruhe, Germany) at 333 K in D_2_O. The ^1^H spectrum was referenced with the internal TSP resonance (δH = 0 ppm). The ^13^C spectrum was referenced with the internal TSP ^1^H resonance, following IUPAC recommendations applying the xiref command in Bruker Topspin 4.1.3 software (δC of TSP was around −2.67 ppm after this procedure). For the signal assignments, COSY, HSQC, TOCSY, and HSQC-TOCSY spectra were used. TOCSYs were recorded with MLEV block and 60 msec mixing time. Polysaccharide sample (15 mg) was dissolved in 1 mL of 99.9% D_2_O, treated in an ultrasonic bath for 30 min, freeze-dried, and then dissolved in 600 µL of 99.96% D_2_O and transferred to an NMR-tube.

### 4.8. Absorption Spectra of Iodine Complexes

The iodine staining spectra of glucans were examined using a Spectronic Genesis 5 spectrophotometer (Rochester, NY, USA) under the conditions described by Archibald et al. [41]. All experiments were carried out using freshly prepared solutions, in which the ratio of iodine to potassium iodide was 1:10. Test solutions were prepared containing 2.5 mg of **NP-H**, rabbit muscle glycogen, or potato amylopectin, 2.5 mL of a 0.2% solution of iodine in 2.0% aqueous potassium iodide, and 1 drop of 3 N hydrochloric acid in a total volume of 25 mL half-saturated ammonium sulfate. Spectra of these three solutions had λ_max_ values of 403, 472, and 559 nm, corresponding to average chain lengths of 5, 13, and 23, respectively. Values of ACL were calculated according to the equation: ACL = 16 + 0.114 (λ_max_ − 500) [41,42].

## 5. Conclusions

A high-molecular α-d-glucan **NP** was isolated from the eggs of the sea urchin *Tripneustes gratilla* and investigated by chemical and physicochemical methods. This branched biopolymer was composed of non-reducing terminal, 4-linked, and 4,6-disubstituted Glc residues present in a ratio of 1:8:1, which was proved by methylation analysis. A glycogen-like structure was proposed for **NP** with a linear 4-linked backbone and rather short oligosaccharide branches at position 6. The absence of single Glc branches was proved by analysis of NMR spectra, whereas the presence of long oligomeric branches was excluded by characteristic of **NP**-iodine complex corresponding to chains with ACL of about 5.

The discovery of a highly branched α-d-glucan from *T. gratilla* eggs collected in Vietnamese coastal waters highlights the potential of this edible sea urchin as a valuable marine bioresource. Given the abundance of this species and the structural similarity of **NP** to biologically active α-glucans from other marine organisms, further studies should explore its bioactivities and prospects for development into marine-derived functional or pharmaceutical products. Such research will contribute to the sustainable utilization and value enhancement of *T. gratilla* populations in Vietnam’s coastal ecosystems.

## Figures and Tables

**Table 1:** Composition of **NP** (% *w/w*).

Sample	Glc	Man	Uronic Acids	Sulfate	Protein
**NP**	95.7	1.0	n.d. ^1^	n.d. ^1^	n.d. ^1^

^1^ not detected.

**Table 2:** Methylation analysis of **NP** and commercial amylopectin (molar % of partially methylated alditol acetates).

Residue	PMAA	Relative Percentages	
		NP	Amylopectin
α-Glc*p*-(1→	1,5-Ac_2_-2,3,4,6-Me_4_-Glc	10	4
→4)-α-Glc*p*-(1→	1,4,5-Ac_3_-2,3,6-Me_3_-Glc	80	91
→6]→4)-α-Glc*p*-(1→	1,4,5,6-Ac_4_-2,3-Me_2_-Glc	10	5

**Table 3:** Residues and signals in NMR spectra of **NP**.

Residue		H-1 C-1	H-2 C-2	H-3 C-3	H-4 C-4	H-5 C-5	H-6 C-6
A	→4)-α-Glc*p*-(1→4)	5.37	3.65	3.96	3.64	3.84	3.88, 3.80
		99.96	71.77	73.46	77.60	71.5	60.82
B	α-Glc*p*-(1→4)	5.36	3.60	3.70	3.43	3.73	3.87, 3.76
		100.10	72.01	73.19	69.71	72.92	60.91
C	→4)-α-Glc*p*-(1→6)	4.96	3.57	4.01	3.64	n.d. ^1^	n.d. ^1^
		98.81	72.01	73.46	77.60	n.d. ^1^	n.d. ^1^
D ^2^	→4)-α-Glc*p*-(1→OH	5.23	3.57	3.97	3.64	3.94	n.d. ^1^
		92.05	71.60	73.30	77.82	70.28	n.d. ^1^
E ^2^	→4)-β-Glc*p*-(1→OH	4.65	3.28	3.76	3.64	3.59	n.d. ^1^
		95.95	74.22	76.28	77.60	74.77	61.0

^1^ Exact position of this signal cannot be established due to strong overlap with major component signals and correlations. ^2^ D and E designate two anomeric forms of glucose residues at the reducing ends of molecules.

## Data Availability

All data generated or analyzed during this study are included in this published article. Any additional data will be available upon request.

## Supplementary Materials

The following supporting information can be downloaded at: https://www.mdpi.com/article/10.3390/ijms262110326/s1.

## Abbreviations

The following abbreviations are used in this manuscript:

CSDB	Carbohydrate Structure Database
ACL	Average chain length
PMAA	Partially methylated alditol acetate
TSP	3-(Trimethylsilyl)propionic-2,2,3,3-d_4_ acid sodium salt
BSA	Bovine serum albumin
ROS	Reactive oxygen species
