# Supplementary material for: Chemical Structure of a Branched α-d-Glucan from the Eggs of Sea Urchin Tripneustes gratilla
Source: Int J Mol Sci. 2025 Oct 23;26(21):10326. doi: 10.3390/ijms262110326 (PMC12609645; doi:10.3390/ijms262110326)
Supplement: Supplementary file 1 [file ijms-26-10326-s001.zip › ijms-3901761-supplementary.pdf]

Chemical structure of a branched α-D-glucan from the eggs of sea urchin *Tripneustes gratilla*

Maria I. Bilan[1], Dmitry A. Argunov[1], Vladimir I. Torgov[1], Andrey S. Dmitrenok[1], Dinh Thanh Trung[1], **Thinh Duc Pham[2], Hang Thi Thuy Cao[2], Anatolii I. Usov[1], Nikolay E. Nifantiev[1]***

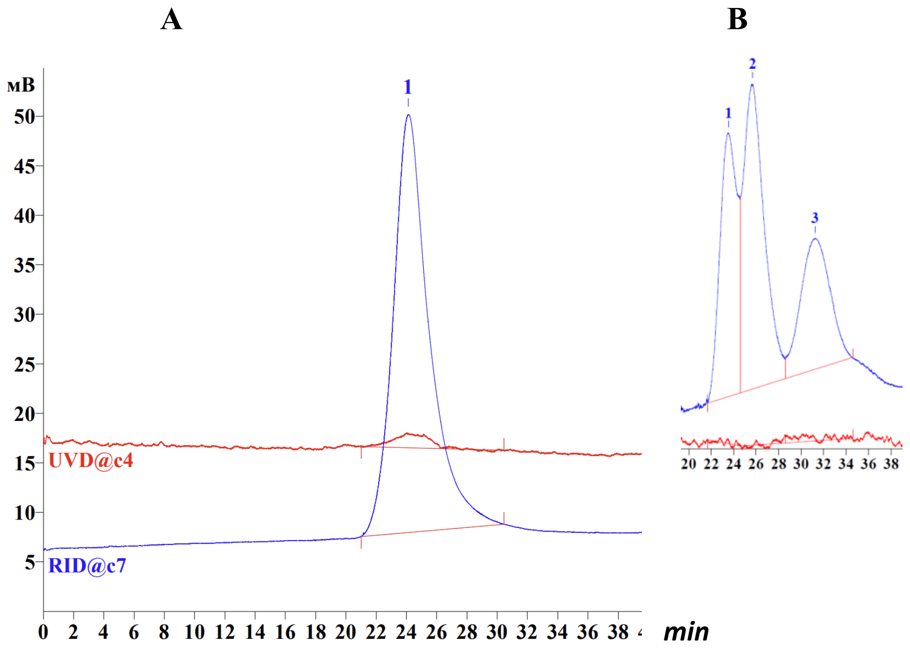

Figure S1. High-performance gel permeation chromatography (HPGPC) profiles.

A. **NP-H** (24.14 min).

B. Standard pullulans:

1). pullulan 805 kDa (23.5 min)

2). pullulan 200 kDa (25.64 min)

3). pullulan 48.8 kDa (31.2 min).
